# Biobox: a toolbox for biomolecular modelling

**DOI:** 10.1093/bioinformatics/btab785

**Published:** 2021-11-15

**Authors:** Lucas S P Rudden, Samuel C Musson, Justin L P Benesch, Matteo T Degiacomi

**Affiliations:** Department of Physics, Durham University, DurhamDH1 3LE, UK; Department of Physics, Durham University, DurhamDH1 3LE, UK; Department of Chemistry, Biochemistry Building, University of Oxford, Oxford OX1 3QU, UK; Department of Physics, Durham University, DurhamDH1 3LE, UK

## Abstract

**Motivation:**

The implementation of biomolecular modelling methods and analyses can be cumbersome, often carried out with in-house software reimplementing common tasks, and requiring the integration of diverse software libraries.

**Results:**

We present Biobox, a Python-based toolbox facilitating the implementation of biomolecular modelling methods.

**Availability and implementation:**

Biobox is freely available on https://github.com/degiacom/biobox, along with its API and interactive Jupyter notebook tutorials.

## 1 Introduction

**Fig. 1.**
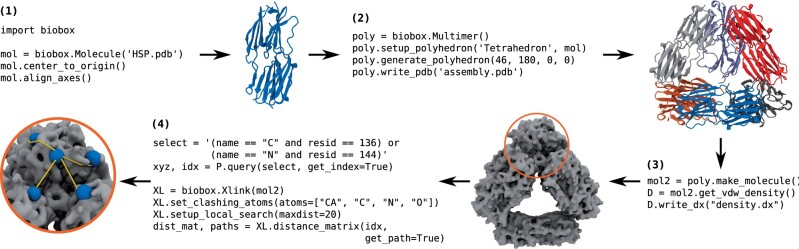
Example of biomolecular data manipulation with Biobox: (1) import a protein structure, (2) generate a tetrahedral scaffold and assemble protein subunits along its vertices, (3) simulate an approximate electron density based on the assembly and (4) identify and measure the length of solvent-accessible paths between residues of interest

Models rationalizing sparse and low-resolution information on biomolecular structure, dynamics and interactions can provide key insight into biological function at the atomic level. Such models are generally produced by exploiting or combining collections of available molecular structures so as to recapitulate experimental observables, and can then be used to predict quantities or properties hard to determine experimentally. A software package handling all common operations within a typical modelling problem would simplify the implementation of custom computational tools. This package should facilitate the simulation of experimental observables, account for the possibility of multiple molecular conformations, accommodate different molecular representations (atomistic, coarse-grained, volumetric) and interface with established scientific computing packages. We found that existing software suites such as MDAnalysis (Michaud-Agrawal *et al.*, 2011), Integrative Modeling Platform (Russel *et al.*, 2012) and Molecular Modeling Toolkit (Hinsen, 2000), though powerful for their target applications, did not fully suit all our requirements. With these focussed on molecular simulations trajectory analysis, highly specific biomolecular modelling problems, or possessing incompatibility with Python >2.7, respectively, a more generalizable, yet easy-to-use module was essential for our applications. To meet our needs, we therefore developed Biobox, a Python package that underpins much of our molecular modelling work. We have made Biobox available along with detailed documentation and tutorials, to those seeking a simple Python toolkit facilitating both the pre- and post-processing of general biomolecular modelling tasks. Hereafter, we present Biobox for the first time, and illustrate its main features by summarizing recent published research featuring its usage (example in Fig.  1). 

## 2 Approach

Biobox manipulates collections of point clouds. Given a system of *N* points, their positions are stored as a 3D NumPy array (van der Walt *et al.*, 2011) of shape (*M*, *N*, 3), where *M* is a dimension corresponding to alternative coordinates. Biobox feature methods to transform electron densities into point clouds and vice versa, and to generate point spatial arrangements respecting predefined shapes and symmetries. Optional metadata associated with each point can be stored in an expandable Pandas (McKinney, 2010) DataFrame. A flexible molecule is therefore a collection of alternative 3D atomic coordinates, stored with metadata information on each atom’s properties and hierarchy (residue, chain). Thus, Biobox leverages on Pandas indexing features to select atoms of interest and, through NumPy, enables direct access to advanced data analysis features within popular scientific computing packages (Harris *et al.*, 2020). Besides quantities directly measurable from point positions and dynamics (e.g. interatomic distances or root mean square fluctuations), quantities such as collision cross sections [CCS, via IMPACT (Marklund *et al.*, 2015)], small-angle X-ray scattering [via Crysol (Franke *et al.*, 2017)] and chemical cross-linking [implementing our accurate DynamXL method (Degiacomi *et al.*, 2017)] can be simulated.

## 3 Applications

Protein–protein docking is the prediction of how proteins of known atomic structure assemble in specific complexes. The exploration of the complex landscape describing all possible protein arrangements is complicated by the fact that proteins are not rigid structures. Our blind protein–protein docking engine, JabberDock (Rudden and Degiacomi, 2019), predicts dimeric arrangements by leveraging a novel molecular representation that encompasses protein electrostatics, shape and local dynamics. JabberDock has been extended to transmembrane protein docking (Rudden and Degiacomi, 2021), and applied to the prediction of the *bo*_3_ oxidase dimeric structure (Olerinyova *et al.*, 2021) by leveraging mass photometry data. Biobox forms the cornerstone of JabberDock by handling the importing and exporting of protein structures and volumetric representations, and manipulating them during the docking process.

Many proteins combine into complexes larger than dimers. Biobox enables the creation of arbitrarily large oligomers and provides the means to impose specific symmetries on the assembly. In particular, Biobox enables assembling molecules according to polyhedral symmetries via a method first adopted by Baldwin *et al.* (2011). In this method, polyhedra are treated like deformable scaffolds upon which monomers can be aligned and roto-translated either individually or in concert. When building any assembly, symmetric or not, multiple models can be appended as alternative conformations, facilitating their comparison (e.g. clustering). The macromolecular assembly methods of Biobox have been leveraged to demonstrate that the small heat-shock protein (HSP) 16.9 forms tetrahedral assemblies (Santhanagopalan *et al.*, 2018). This required systematically building hexamers of HSP16.9 dimeric building block according to all possible symmetries, then selecting only those that both satisfied the experimentally determined CCS and allowed the binding of C-terminal inter-dimer linkers modelled as solvent-accessible paths via our DynamXL method (Degiacomi *et al.*, 2017). In another application, Biobox helped demonstrate that the Spa33-FL/C2 injectisome basal body subcomplexes detected by mass spectrometry were assembled into chains (Mcdowell *et al.*, 2016). Since a section of the assembly subunit’s atomic structure was unknown, we built super-coarse-grained models, where each protein was treated as an ellipsoid-shaped point cloud. We could demonstrate that experimental CCS measures were consistent with these subunits being assembled into chains of different lengths, as opposed to an aggregate. Another application involving CCS calculations of super-coarse-grained models involved the determination of ideal sphere-overlap levels in the context of protein assembly modelling, where each subunit is represented as a single, large sphere (Degiacomi, 2019).

The examples above demonstrate how Biobox enables calculating CCS values of both atomic and super-coarse-grained models. A further extension to this is its capability to estimate the CCS of electron densities by implementing the EM∩IM method (Degiacomi and Benesch, 2016). In EM∩IM, the most suitable map isovalue is identified based on knowledge of protein mass and map resolution. Besides providing a means to define map contours, resulting in a representative visualization of data as a density map, the CCS of the resulting volume itself can be explicitly calculated by transforming it into a dense point cloud. Biobox also enables the opposite operation, i.e. transforming a point cloud into a density map. This feature was used to study the interactions within a molecular dynamics simulation of the Na^+^/H^+^ antiporter (NapA) embedded in a lipid bilayer (Landreh *et al.*, 2017). The CCS of protein-lipid pairs extracted from the simulation were calculated, enabling the identification of lipid arrangements recapitulating experimental data. To represent data, we transformed the coordinates of all phosphate atoms into a 3D probability density, saved via Biobox in OpenDX format for ease of visualization in molecular graphics software.

Overall, Biobox facilitates the development of biomolecular modelling methods by handling much of the complex yet necessary pre-processing and molecular structure manipulation tasks in a few simple lines of code.
